# Effectiveness and safety of a model predictive control (MPC) algorithm for an artificial pancreas system in outpatients with type 1 diabetes (T1D): systematic review and meta-analysis

**DOI:** 10.1186/s13098-022-00962-2

**Published:** 2022-12-09

**Authors:** Su Lim Kang, Yoo Na Hwang, Ji Yean Kwon, Sung Min Kim

**Affiliations:** 1 Department of Medical Device and Healthcare, Dongguk University-Seoul, 26, Pil-Dong 3-Ga, Seoul, Jung-Gu 04620 Republic of Korea; 2 Department of Medical Device Regulatory Science, Dongguk University-Seoul, 26, Pil-dong 3-Ga, Seoul, Jung-Gu 04620 Republic of Korea

**Keywords:** Artificial pancreas, Algorithm, Model predictive control, Hypoglycemia, Type 1 diabetes

## Abstract

**Background:**

The purpose of this study was to assess the effectiveness and safety of a model predictive control (MPC) algorithm for an artificial pancreas system in outpatients with type 1 diabetes.

**Methods:**

We searched PubMed, EMBASE, Cochrane Central, and the Web of Science to December 2021. The eligibility criteria for study selection were randomized controlled trials comparing artificial pancreas systems (MPC, PID, and fuzzy algorithms) with conventional insulin therapy in type 1 diabetes patients. The heterogeneity of the overall results was identified by subgroup analysis of two factors including the intervention duration (overnight and 24 h) and the follow-up periods (< 1 week, 1 week to 1 month, and > 1 month).

**Results:**

The meta-analysis included a total of 41 studies. Considering the effect on the percentage of time maintained in the target range between the MPC-based artificial pancreas and conventional insulin therapy, the results showed a statistically significantly higher percentage of time maintained in the target range in overnight use (10.03%, 95% CI [7.50, 12.56] p < 0.00001). When the follow-up period was considered, in overnight use, the MPC-based algorithm showed a statistically significantly lower percentage of time maintained in the hypoglycemic range (−1.34%, 95% CI [−1.87, −0.81] p < 0.00001) over a long period of use (> 1 month).

**Conclusions:**

Overnight use of the MPC-based artificial pancreas system statistically significantly improved glucose control while increasing time maintained in the target range for outpatients with type 1 diabetes. Results of subgroup analysis revealed that MPC algorithm-based artificial pancreas system was safe while reducing the time maintained in the hypoglycemic range after an overnight intervention with a long follow-up period (more than 1 month).

**Supplementary Information:**

The online version contains supplementary material available at 10.1186/s13098-022-00962-2.

## Background

Type 1 diabetes (T1D), which occurs mainly in children and adolescents, is caused by insulin deficiency due to the auto-immune destruction of beta cells in the pancreas [1]. T1D patients require intensive management early in the disease to achieve HbA1c levels close to normal [2]. The biggest problem with T1D management is the occurrence of severe hypoglycemia and ketoacidosis [3]. In particular, hypoglycemia is known as a symptom that occurs frequently in patients with T1D due to inadequate dosage and timing of sulfonylureas or insulin used for treatment. [4]. Continuous blood glucose monitoring and insulin dose adjustment are keys to reducing such complications [5]. Clinical research on insulin therapy is needed for effective treatment and the prevention of complications. In addition, studies according to the follow-up period are necessary to obtain clinical evidence for identifying the risk factors [6].

Conventional insulin therapy includes continuous subcutaneous insulin infusion (CSII) and sensor-augmented pumps (SAP). CSII is a treatment that delivers insulin at a preselected basal infusion rate by the sensor. It is not administered automatically according to blood glucose. Instead, it is injected by calculating and setting the dose according to manually measured blood sugar level. It does not provide blood glucose data. BG measurements are used to make treatment decisions [7]. SAP consists of a combination of CGM and CSII, a glucose sensor introduced into insulin pump therapy [8, 9]. This is capable of monitoring treatment and response of the patients [10]. CSII and SAP have the advantage of being able to control the infusion rate as much as needed and continuously deliver insulin. However, the disadvantage is that if the pump does not work properly, the risk of complications by administering too little or too much insulin can be high [11]. To compensate for these shortcomings, artificial pancreas systems have been developed. The real strength of the artificial pancreas system is in regulating basal insulin injections to change the glycemic status (both hyperglycemia and hypoglycemia). That is, improving metabolic control without increasing the risk of hypoglycemia in T1D patients by aiming to increase the proportion of time in the target range [12].

An artificial pancreas is an automated system that automatically measures blood glucose levels to achieve the target range and injects insulin into the blood accordingly. This system consists of three devices including: (1) a sensor, such as a continuous glucose monitor (CGM) that transmits data to an algorithm after measuring blood glucose; (2) an algorithm that analyzes the data and calculates the required insulin injection dose, and (3) an insulin infusion pump that delivers insulin according to the algorithm [13]. Among these, at the core of artificial pancreas technology is an algorithm that calculates the amount of insulin required to maintain a patient's glucose level within the target range [14]. There are three main types of control algorithms for glucose regulation, model predictive control (MPC), proportional integral derivative (PID), and fuzzy logic (FL). The MPC algorithm predicts future glucose levels to bring current blood glucose levels into the target range. The PID algorithm analyzes the deviation of the measured glucose from the target range to calculate the amount of insulin to deliver. The fuzzy algorithm quickly mimics the insulin dose calculates made by clinical experts based on monitoring data [15]. The main role of an algorithm is to keep blood glucose level in a safe range. Therefore, the type of algorithm is considered an important factor in safe blood glucose control.

Several meta-analyses have compared artificial pancreas systems and conventional insulin therapy according to the intervention period [16–18] and, hormone type [16, 17]. *Weisman *et al [16] and *Karageorgiou *et al [19] evaluated the effectiveness and safety of artificial pancreas algorithm types, but reported no details regarding which algorithms affected the outcomes. In a recent clinical study (*Haidar *et al. [20]), an MPC-based artificial pancreas system (69%) showed a greater effect on the percentage of time maintained in the target blood glucose range than a sensor-augmented pump (61%). *Pinsker* and colleagues directly compared the effectiveness of artificial pancreas systems according to algorithm types (MPC vs PID) with conventional insulin therapy [21]. The mean difference was greater for the MPC (74.4%) than for the PID algorithm (63.7%). To the best of our knowledge, no previous meta-analysis has analyzed the influence of algorithm types on outcomes and compared them according to the follow-up period. This systematic review and meta-analysis aimed to determine whether an MPC algorithm-based artificial pancreas system might be more effective and safe than conventional insulin therapy in terms of the risk of hypoglycemia and maintaining glucose levels within the target range in outpatients with T1D. Moreover, to identify the finding of the previous studies [16, 21] that the MPC algorithm performed well or better than PID in terms of safe and effective glucose management, additional meta-analysis has analyzed the influence of algorithm types on outcomes.

## Methods

### Search strategy

We searched PubMed, EMBASE, Web of Science (WoS), and the Cochrane Central Register of Controlled Trials to December 2021. For the search terms, mesh terms and natural languages were used, including: “((((artificial pancreas[MeSH Terms]) OR (diabetes mellitus[MeSH Terms])) AND (diabetes mellitus, type 1[MeSH Terms])) AND (controlled clinical trials, randomized[MeSH Terms])) AND (algorithm[MeSH Terms])”, “Artificial pancreas OR Closed-loop system AND Diabetes mellitus type 1 AND Clinical trial AND Randomized clinical trial”. This study was reported according to the Preferred Reporting Items for Systematic Review and Meta-Analyses (PRISMA) statement (PROSPERO, number 2021:CRD42021268271) (PRISMA Checklist–Additional file 1: Table S1).

### Inclusion and exclusion criteria

Studies that met the following criteria were included: (1) type 1 diabetes outpatients including non-pregnant adults, children, and infants; (2) interventions using an algorithm-based artificial pancreas; (3) comparisons using conventional insulin therapy (SAP or insulin pumps); (4) outcomes including the outcome variables of the percentage of time maintained in the target blood glucose range (3.9–10 mmol), the percentage of time maintained in the hypoglycemic range (< 3.9 mmol), and the daily insulin dose; (5) Randomized clinical trials (RCTs) including crossover and parallel studies. Studies meeting the following criteria were excluded: (1) non-RCT studies; (2) duplicated documents; (3) review papers or letters; (4) clinical trial protocol studies, and (5) studies that did not report at least one of the outcomes.

### Selection of studies

Two reviewers (Kang and Hwang) selected the studies. The studies were screened by title and then selected by abstract and content. Disagreements regarding the original study were discussed, and jointly reviewed to reach a consensus. In the meta-analysis, only specified studies for each purpose were selected from included studies. In the meta-analysis to assess the effectiveness and safety of the MPC algorithm, only studies comparing the MPC-based artificial pancreas system with conventional insulin therapy were selected from included studies. In additional analysis of algorithm types, studies comparing algorithm-based artificial pancreas with conventional insulin therapy were selected from included studies.

### Data extraction

Data including the study, year, patients, devices, intervention, comparator, duration, and follow-up period were extracted. A standardized format was used by two independent reviewers (Kang and Hwang). Disagreements regarding the original study were discussed, and jointly reviewed to reach a consensus.

### Quality assessment

Risk of bias (RoB) is the likelihood that a characteristic of the study design or study conduct will give erroneous results. The RoB is evaluated according to random sequence generation, allocation concealment, blinding of the participants and personnel, blinding of the outcome assessment, incomplete outcome data, selective reporting, and other biases. It was assessed as high, low, or unclear using the Cochrane risk of bias tool.

### Outcome measures

The primary outcome was the percentage of time maintained in the target blood glucose range (3.9–10 mmol). The secondary outcomes were the percentage of time maintained in the hypoglycemic range (< 3.9 mmol) and the daily insulin dose. If the primary and second outcome data were reported separately, they were analyzed separately in this study. Studies done over a 24 h period reported 24 h results and studies done overnight reported overnight results. If the adult and pediatric data were reported separately, they were analyzed separately, and if the single and dual hormone data were reported separately, they were analyzed separately.

### Statistical analysis

The forest plot is one of the most useful tools for providing a visual summary of the analysis results. It graphically presents estimates of the overall effect size and confidence intervals of the included studies. The mean difference (MD) was calculated for all results, and a random-effects model was used. Ninety-five percent confidence intervals (CI) were calculated for all analyses, and the significance level was 0.05 (p < 0.05). When presented as an interquartile range (IQR) value, the standard deviation (SD) was calculated as (*q*3-*q*1)/1.35 according to Cochrane’s recommendation [22, 23]. The heterogeneity was evaluated by *I*^2^. If it was 50% or more, heterogeneity was identified, and 75% or more, heterogeneity was identified as high. Subgroup analysis was conducted to identify heterogeneity according to the intervention duration (overnight and 24 h), follow-up period (< 1 week, 1 week to 1 month, and > 1 month), and algorithm types (MPC, PID, and Fuzzy). Sensitivity analysis was performed on the primary outcome, which was the percentage of time maintained in the target blood glucose range to explore the cause of high heterogeneity. Meta-analysis was analyzed using Revman 5.3 software, and statistical analysis was performed through SPSS Statistics 25.

## Results

### Characteristics of the included studies

**Fig. 1:**
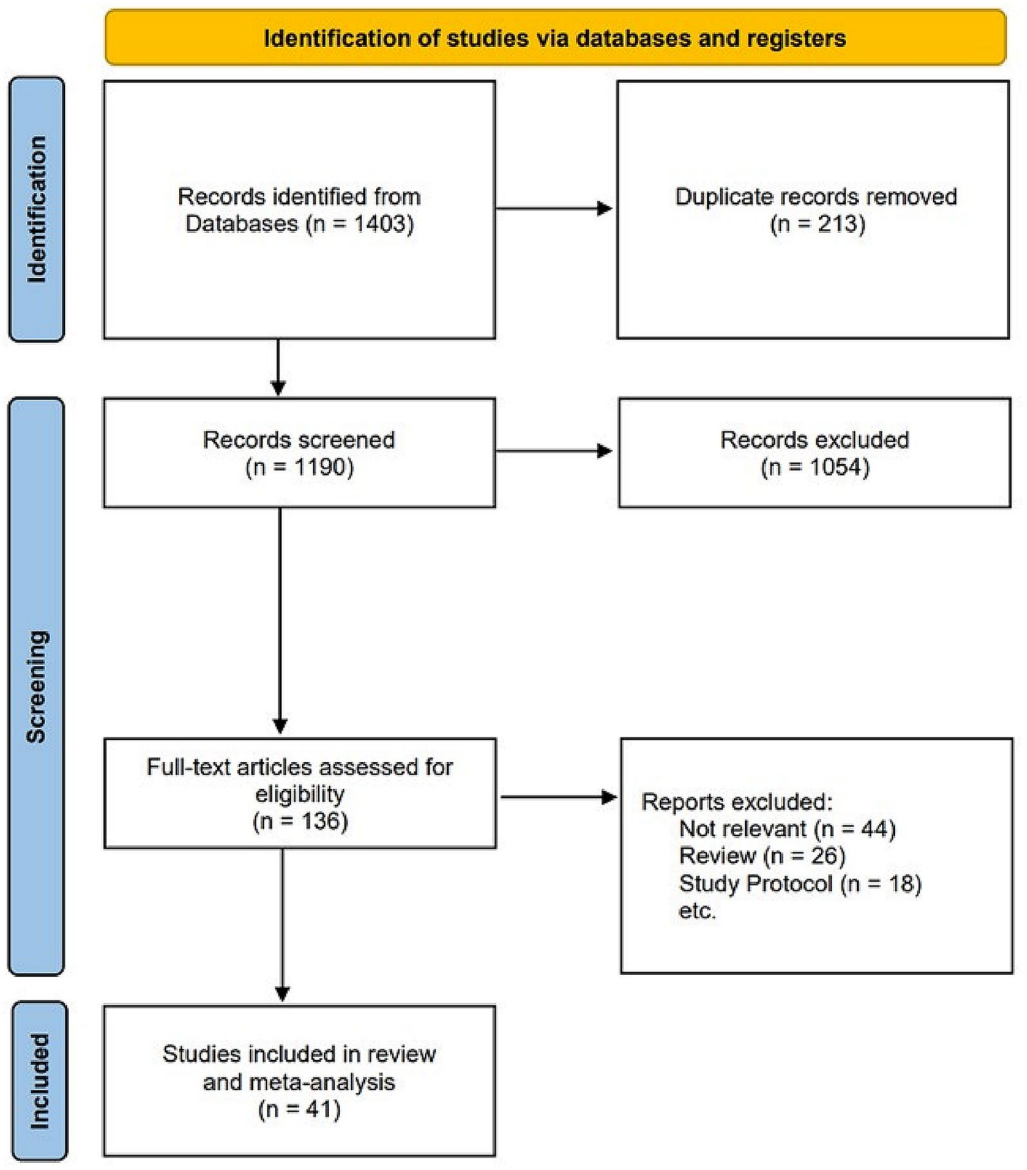
Flowchart of the study selection

Figure 1 shows a flowchart of the study selection process. A total of 1,403 studies were searched, 213 duplicate studies were removed and 1,190 studies were screened. Of the studies, 1,054 were excluded by the title and abstract contents, and the full-text of 136 studies was evaluated. Unrelated documents such as reviews, letters, and clinical trial protocols were excluded. Most clinical trials performed artificial pancreas system (MPC, PID, and Fuzzy) vs. conventional insulin therapy. To analyze the effectiveness and safety of MPC algorithm-based artificial pancreas systems and the influence of algorithm type on outcomes, inclusion criteria were set as studies that compared artificial pancreas system with conventional insulin therapy. A total of 41 documents were finally included.

Table 1 shows the characteristics of 41 studies [20, 24–62] included in the systematic review (1,398 total patients). Nine trials compared insulin pumps, and 32 trials compared sensor-augmented pumps. Thirty-three trials used the MPC algorithm, 6 trials used the PID algorithm, and 2 trials used the fuzzy algorithm. The artificial pancreas system was used overnight in 15 trials and 24 h in 26 trials. According to the follow-up period, 18 trials reported periods of less than 1 week, 14 trials reported between 1 week and 1 month, and 9 trials reported periods of more than 1 month. The detailed characteristics are given in Additional file 1: Table S2.

**Table 1:** Characteristics of the included studies

No	Study	Year	Patients	Intervention	Algorithm	Comparator	Duration	Follow-up
1	Anderson et al. [24]	2019	42	DiAs USS with Dexcom	MPC	SAP	24 h	4 weeks
2	Bally et al. [25]	2017	29	Florence with freestyle navigator	MPC	SAP	24 h	4 weeks
3	Benhamou et al. [26]	2019	63	Hybrid closed-loop system	MPC	SAP	24 h	12 weeks
4	Blauw et al. [27]	2016	10	Inreda diabetic	PID	Pump	24 h	4 days
5	Breton et al. [28]	2020	101	t:slim X2 insulin pump, Dexcom with Control-IQ Technology	MPC	SAP	24 h	16 weeks
6	Breton et al. 2 [29]	2017	32	t:AP pump or Roche Accu-Chek Spirit Combo pump, Dexcom with DiAs	MPC	SAP	24 h	120 h
7	Brown et al. [30]	2019	168	t:slim X2 insulin pump with Control-IQ Technology, Tandem Diabetes Care, Dexcom	MPC	SAP	24 h	6 months
8	Brown et al. 2 [31]	2017	40	DiAs USS with Dexcom	MPC	SAP	Overnight	5 days
9	Brown et al. 3 [32]	2015	10	Accu-Chek Spirit Combo pump or personal pump, Dexcom with DiAs system	PID	SAP	Overnight	5 days
10	Chernavvsky et al. [33]	2016	16	DiAs USS with Dexcom	MPC	Pump	24 h	1 day
11	De bock et al. [34]	2018	12	Medtronic MiniMed Hybrid Closed Loop System	MPC	Pump	24 h	7 days
12	De boer et al. [35]	2017	12	DiAs USS with Dexcom	MPC	SAP	24 h	3 days
13	Del Favero et al. [36]	2016	30	Accu-Chek Spirit Combo pump or personal pump, Dexcom with DiAs system	MPC	SAP	24 h	72 h
14	El-Khatib et al. [37]	2017	39	Two(one for insulin, one for glucagon) t:Slim infusion pumps, Dexcom	MPC	Pump	24 h	11 days
15	Elleri et al. [38]	2013	12	SEVEN PLUS; Dexcom	MPC	Pump	Overnight	36 h
16	Forlenza et al. [39]	2017	19	DiAs	MPC	SAP	24 h	2 weeks
17	Forlenza et al. 2 [40]	2017	28	Medtronic PHHM	MPC	SAP	Overnight	21 nights
18	Haidar et al. [20]	2021	36	Dexcom CGM system, t:slim TAP3 insulin pump	MPC	SAP	24 h	12 days
19	Hovorka et al. [41]	2014	16	Florence with FreeStyle Navigator	MPC	SAP	Overnight	21 days
20	Huyett et al. [42]	2017	10	DiAs with Dexcom	MPC	SAP	24 h	72 h
21	Kovatchev et al. [43]	2020	125	Accu-Chek Spirit Combo insulin pump, Dexcom CGM system, and inControlAP	MPC	SAP	Overnight	3 months
22	Kovatchev et al. 2 [44]	2020	78	Accu-Chek Spirit Combo insulin pump, Dexcom CGM system, and inControlAP	MPC	SAP	Overnight	10 months
23	Kovatchev et al. 3 [45]	2014	18	Tandem t:slim pump, with DiAs system	PID	SAP	24 h	40 h
24	Kropff et al. [46]	2015	32	Accu-Chek Spirit Combo insulin pump, Dexcom CGM system	MPC	SAP	Overnight	12 weeks
25	Leelarathna et al. [47]	2014	17	Florence with FreeStyle Navigator	MPC	SAP	24 h	8 days
26	Ly et al. [48]	2016	21	Medtronic MiniMed Hybrid Closed Loop System	PID	SAP	Overnight	5–6 days
27	Ly et al. 2 [49]	2015	21	Medtronic MiniMed Hybrid Closed Loop System	PID	SAP	24 h	6 days
28	Ly et al. 3 [50]	2014	20	Medtronic MiniMed Hybrid Closed Loop System	PID	SAP	Overnight	5–6 days
29	Nimri et al. [51]	2014	24	MD-Logic system with Medtronic Paradigm Veo pump	Fuzzy	SAP	Overnight	6 weeks
30	Nimri et al. 2 [52]	2014	15	MD-Logic system with Medtronic Paradigm Veo pump	Fuzzy	SAP	Overnight	4 days
31	Renard et al. [53]	2018	23	DiAs with Dexcom	MPC	SAP	24 h	2 days
32	Russell et al. [54]	2016	19	Two(one for insulin, one for glucagon) t:Slim infusion pumps, Dexcom	MPC	Pump	24 h	5 days
33	Russell et al. 2a [55]	2014	20	Two(one for insulin, one for glucagon) t:Slim infusion pumps, Dexcom	MPC	Pump	24 h	5 days
34	Russell et al. 2b [55]	2014	32	Two(one for insulin, one for glucagon) t:Slim infusion pumps, Dexcom	MPC	Pump	24 h	5 days
35	Sherr et al. [56]	2020	11	Omnipod hybrid closed loop system	MPC	Pump	Overnight	7 days
36	Spaic et al. [57]	2017	30	Medtronic PHHM	MPC	SAP	Overnight	21 nights
37	Tauschmann et al. [58]	2018	86	Medtronic hybrid closed loop system	MPC	SAP	24 h	12 weeks
38	Tauschmann et al. 2 [59]	2016	12	Florence with freestyle navigator	MPC	SAP	24 h	3 weeks
39	Tauschmann et al. 3 [60]	2016	12	Florence with freestyle navigator	MPC	SAP	24 h	7 days
40	Thabit et al. [61]	2015	33	Florence with freestyle navigator	MPC	SAP	24 h	12 weeks
41	Thabit et al. 2 [62]	2014	24	Florence with freestyle navigator	MPC	SAP	Overnight	4 weeks

*MPC* Model Predictive Control, *PID* Proportional Integral Derivative, *SAP* Sensor-Augmented Pump, *CGM* Continuous Glucose Monitoring, *DiAs* Diabetes Assistant

### Assessment of risk of bias

The results of the bias evaluation are presented in Additional file 1: Figures S1, S2. Of the 41 studies, only 13 studies had a low risk, and 4 studies showed a high risk. Three of 4 studies were rated at high risk due to insufficient data. One was at high risk due to the small number of patients and lack of patient information.

### Primary outcome

Thirty-three comparisons with 1,311 patients were pooled to analyze the effectiveness of glucose control of the MPC-based artificial pancreas system. The percentage of time maintained in the target blood glucose range was 12.57% ([MD], 95% CI [9.63, 15.50] p < 0.00001), higher than that of, conventional insulin therapy (Additional file 1: Table S3). However, the heterogeneity was high (*I*^2^ = 89%).

### Secondary outcomes

Thirty comparisons with 1,237 patients were pooled to analyze the safety according to time maintained in hypoglycemia range in the MPC-based artificial pancreas system. The percentage of time maintained in the hypoglycemic range was −1.12% ([MD], 95% CI [-1.50, -0.75] p < 0.00001) in the MPC algorithm-based artificial pancreas system, lower than that with the conventional insulin therapy (Additional file 1: Table S3). However, the heterogeneity still existed (*I*^2^ = 64%).

Sixteen comparisons with 724 patients reported U/day or U/8 h were pooled for evaluating the daily insulin dose in the MPC-based artificial pancreas system. The daily insulin dose in the MPC algorithm-based artificial pancreas system showed a statistically significant decrease in 24 h interventions ([MD], −1.24U, 95% CI [−2.43, −0.06] p = 0.04), compared to conventional insulin therapy (Additional file 1: Table S4).

### Subgroup analysis of intervention timing (24 h versus overnight) in studies using the MPC algorithm

Subgroup analysis was performed to identify the cause of the heterogeneity in the percentage of time maintained in the target blood glucose range and hypoglycemic range, when restricted to studies using the MPC algorithm with sufficient data. The protocol for the subgroup analysis is shown in Additional file 1: Figure S3. We subdivided the studies according to the timing of the intervention (24 h or overnight).

**Fig. 2:**
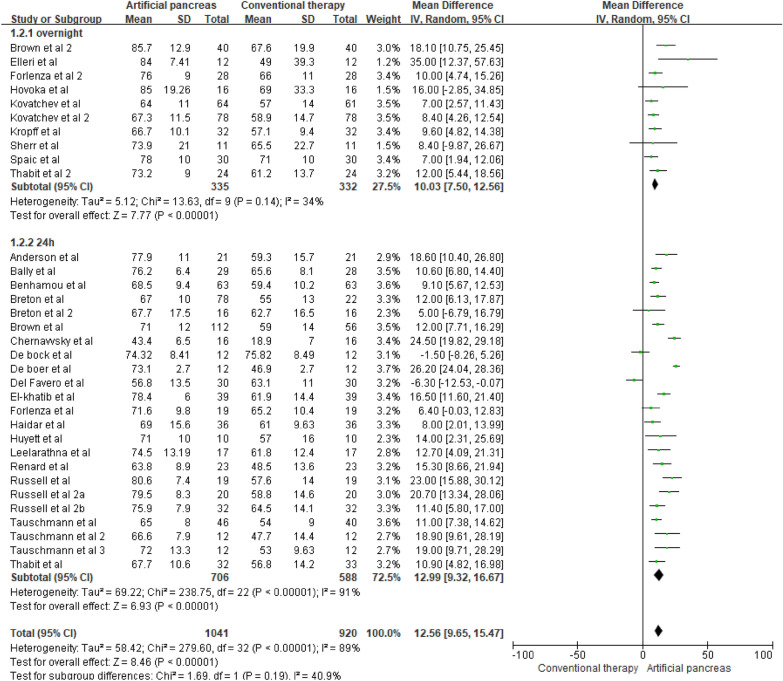
Mean difference in time maintained in the target blood glucose range according to the intervention duration (MPC algorithm-based artificial pancreas)

**Fig. 3:**
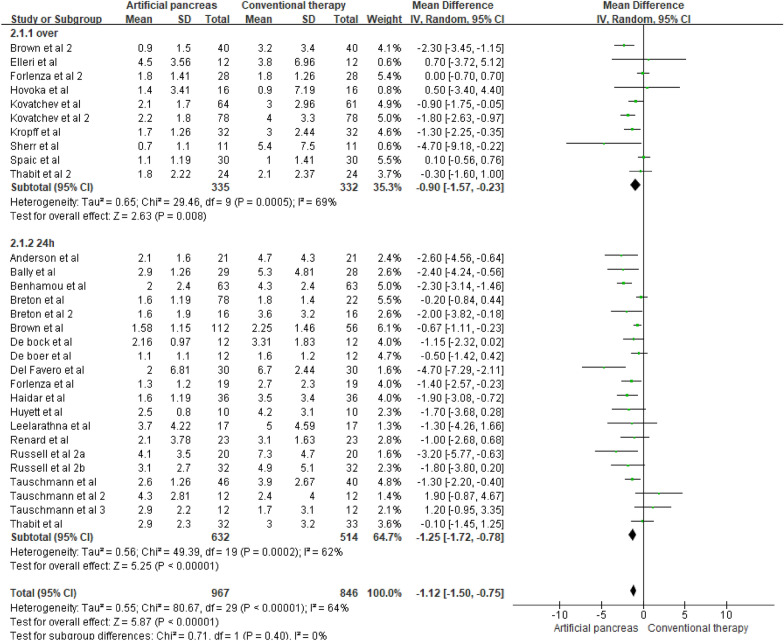
Mean difference in time maintained in the hypoglycemic range according to the intervention duration (MPC algorithm-based artificial pancreas)

Figures 2 and 3 show the forest plots of MPC-based artificial pancreas systems versus conventional insulin therapy for the percentage of time maintained in the target blood glucose range (23 24 h studies and 10 overnight studies) and hypoglycemic range (20 24 h studies and 10 overnight studies), respectively. Differences in the percentage of time maintained in the target blood glucose range were higher in studies with 24 h of intervention ([MD], 12.99%, 95% CI [9.32, 16.67] p < 0.00001) compared to overnight interventions ([MD], 10.03%, 95% CI [7.50, 12.56] p < 0.00001, Fig. 2). However, there was high heterogeneity (*I*^2^ = 91%) in the subgroup of studies with 24 h interventions, which requires further exploration.

Differences in the reductions in hypoglycemia were higher with 24 h of intervention ([MD], −1.25%, 95% CI [−1.72, −0.78] p < 0.00001) compared to overnight interventions ([MD], −0.90%, 95% CI [−1.57, −0.23] p = 0.008, Fig. 3). However, there was moderate heterogeneity between the trials within each of these subgroups (overnight: *I*^2^ = 69%; 24 h: *I*^2^ = 62%). Therefore, the validity of the treatment effect estimate for each subgroup is uncertain, as the individual trial results were inconsistent.

### Subgroup analysis of follow-up periods in studies using the MPC algorithm

**Fig. 4:**
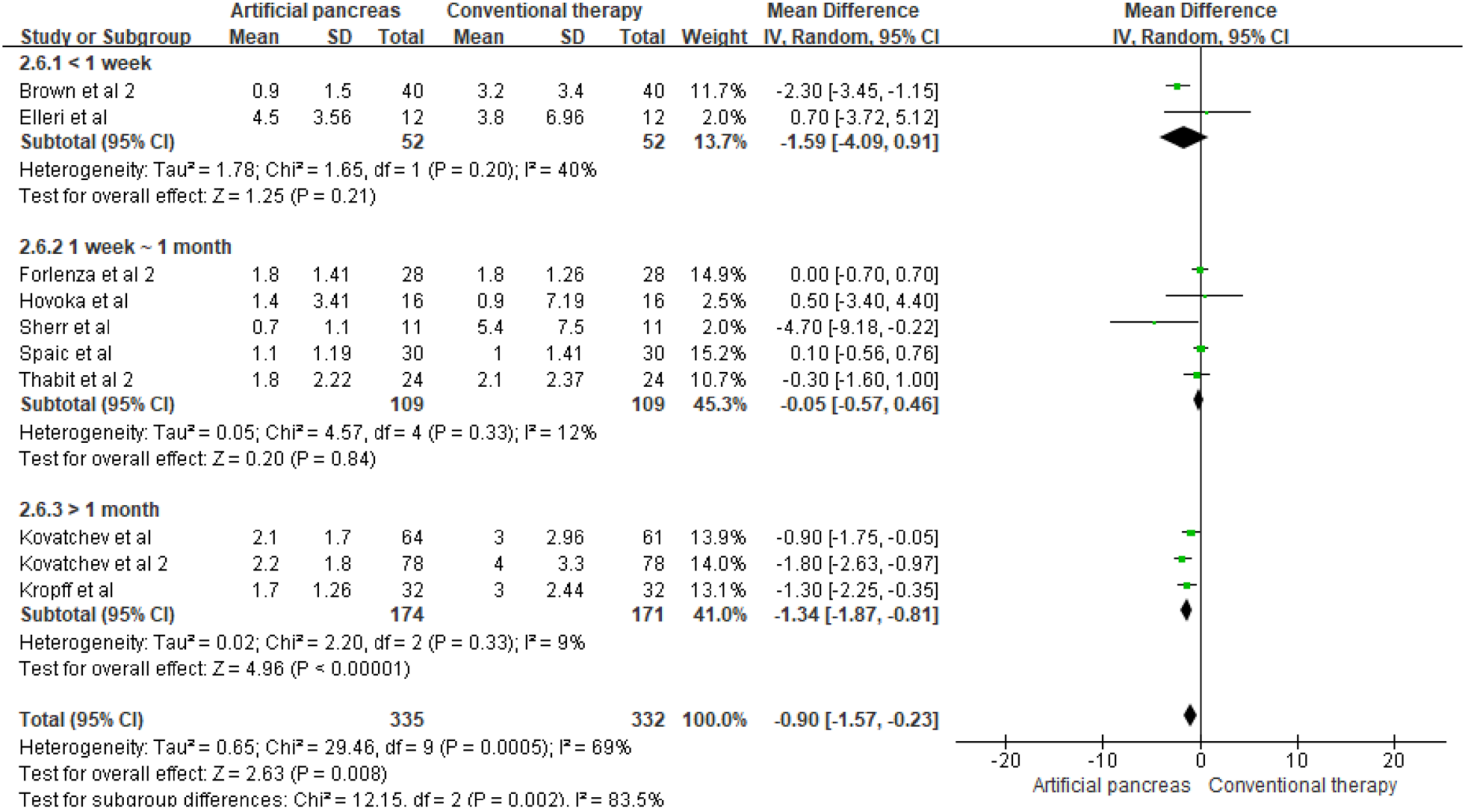
Mean difference in time maintained in the hypoglycemic range according to the follow-up period (artificial pancreas (MPC-overnight))

If there was heterogeneity in the subgroup analysis according to the intervention duration, we subdivided the studies by follow-up period. The percentage of time maintained in the hypoglycemic range in the long-term (G3) group with overnight interventions was −1.34% ([MD], 95% CI [−1.87, −0.81] p < 0.00001, Fig. 4) lower, showing a statistically difference. In contrast, there was no significant difference between these subgroups in the time maintained in the target blood glucose range and hypoglycemic range in studies with 24 h intervention (Additional file 1: Figures S4 and S5).

### Additional analysis of algorithm types (MPC, PID, and Fuzzy) for primary and secondary outcomes

To verify findings of previous studies [16, 21], we performed additional meta-analysis for different algorithm types (MPC, PID, and Fuzzy) compared to conventional insulin therapy. The MPC algorithm-based artificial pancreas system was associated with a higher percentage of time maintained in the target blood glucose range ([MD], 12.57%, 95% CI [9.63, 15.50] p < 0.00001) than the PID algorithm-based artificial pancreas system ([MD], 9.59%, 95% CI [−3.67, 22.85] p < 0.00001). Reductions of hypoglycemia were associated with a higher in studies using the PID ([MD], -5.24%, 95% CI [−16.06, 5.58] p < 0.00001) and Fuzzy ([MD], -20.80%, 95% CI [−64.12, 22.52] p = 0.0007) algorithms than in studies using the MPC ([MD], -1.12%, 95% CI [−1.50, −0.75] p < 0.00001) algorithm. However, studies using the PID algorithm (3 studies, 52 patients) and the fuzzy algorithm (2 studies, 90 patients) had far smaller numbers of trials and participants than studies using the MPC algorithm (30 studies, 1,185 patients), meaning that the analysis was unlikely to produce useful findings (Additional file 1: Table S3).

### Sensitivity analysis

Sensitivity analysis was performed to explore the cause of the high heterogeneity (93%) in the percentage of time maintained in the target blood glucose range (Additional file 1: Figure. S4). In the analysis, only 13 studies with low risk were included, and 28 studies with risk of bias due to insufficient data or unclear results were excluded. The sensitivity analysis, confirmed that heterogeneity was reduced to 32% (Additional file 1: Figure. S6).

### Publication bias

Publication bias was evaluated by a funnel plot (Additional file 1: Figures S7–S9). Studies with a small sample size are grouped at the bottom of the graph, and studies with a large sample size are grouped at the top. If there was publication bias, the overall appearance was asymmetrical. The funnel plot in this study showed a symmetrical pattern, indicating no publication bias.

## Discussion

This study aimed to determine whether an MPC algorithm-based artificial pancreas system might be more effective than conventional insulin therapy in terms of hypoglycemia risk and maintaining glucose levels within the target range in outpatients with T1D. Most clinical trials compared artificial pancreatic systems (MPC, PID, and Fuzzy) and conventional insulin therapy for the percentage of time maintained in the target and hypoglycemic range. Since artificial pancreas systems might be influenced by the algorithm type and follow-up period, a meta-analysis including these variables is required. In previous studies, meta-analyses were performed according to the intervention period [16–18] or hormone type [16, 17]. To the best of our knowledge, no previous meta-analysis has determined effects of algorithm type on outcomes or compared them according to the follow-up period. We hypothesized that the MPC algorithm would have an influence on reducing hypoglycemia. The main result highlights are as follows. The percentage of time maintained in the target range in MPC-based artificial pancreas systems was high when they were used overnight (10.03% [7.50, 12.56] p < 0.00001) and for 24 h (12.99% [9.32, 16.67] p < 0.00001). The percentage of time maintained in the hypoglycemia range in MPC-based artificial pancreas system was low when used overnight and for more than 1 month (−1.34% [−1.87, −0.81] p < 0.00001). Therefore, it is considered that an MPC-based artificial pancreas system could improve glucose control while reducing the risk of hypoglycemia, compared to conventional insulin therapy in long-term (> 1 month) use. Moreover, to verify findings of previous studies [16, 21] showing that the MPC algorithm performed well or better than PID in terms of safe and effective glucose management, we performed additional meta-analysis for different algorithm types compared to conventional insulin therapy. The MPC-algorithm group showed more improvement in the time maintained in the target blood glucose range than the PID algorithm group (p < 0.00001). This finding was consistent with a recent subgroup analysis in a meta-analysis study [16], in which PID algorithms had substantially less improvement in the percentage of time maintained in the target blood glucose range than MPC and fuzzy logic algorithms.

The percentage of time maintained in the target blood glucose range was analyzed to confirm the effect of blood glucose control. Compared to conventional insulin therapy, MPC-based artificial pancreas system showed high percentages of time maintained in the target blood glucose range when used overnight and 24 h. The basic principle of MPC is that the model is used to predict the impact of a control shift on future output, and optimization is performed to select the best set of current and control shifts to achieve the goal. The main advantages of MPC are that (a) restrictions on insulin delivery rates are explicitly controlled in the calculations, (b) a general framework that can include the effects of meals, exercise, and other events that are functions of the time of day, and (c) it is flexible enough to include targets ranging from set points to zones [63]. These characteristics of MPC are considered to have a great impact on improving blood glucose control in T1D patients.

Hypoglycemia is a typical complication of type 1 diabetes management systems. To compare the influence on hypoglycemia according to MPC algorithm, the effect size was analyzed for the percentage of time maintained in the hypoglycemic range. Subgroup analysis was additionally performed according to intervention period and follow-up period to identify the cause of the heterogeneity. The analyses showed that the MPC algorithm-based artificial pancreas systems statistically significantly reduced the time in hypoglycemia during overnight use for more than 1 month. The MPC algorithm has important characteristics of being customizable to the patient. In particular, it is considered to have a significant effect on hypoglycemia because it can learn the details of a patient's daily life (e.g., timing, and the duration and intensity of meals and exercise) to optimize insulin infusions. It is considered that the nature of the MPC algorithm would enable rapid recovery from hypoglycemia even overnight when the risk of hypoglycemia could be pronounced [16]. In addition, an automatic insulin delivery algorithm that can learn and adapt to patients’ growth, development, and lifestyle changes could enable the long-term use of artificial pancreas systems [64]. The MPC algorithm can be a suitable condition for long-term blood glucose control. Therefore, it is considered that the MPC-based artificial pancreas system showed a statistically significant decreased risk of hypoglycemia in patients who used it for a long-time (> 1 month).

Daily insulin dose was analyzed to determine the association with risk factors such as the occurrence of hypoglycemia. The results showed a statistically significant difference between the MPC-based artificial pancreas system and conventional insulin therapy in the 24 h group. MPC has safeguards that can be individualized using the insulin basal rate, insulin sensitivity factor, and the time of action for each patient's insulin [65]. To achieve a target blood glucose level, it uses a preprogrammed rate of injection as an initial estimate of the insulin required. As blood glucose levels increase, MPC steps up insulin infusion but works carefully at suboptimal levels [66]. According to these characteristics, the MPC algorithm seems to be able to achieve safely control blood glucose levels by reducing the daily insulin dose.

This study had some limitations. The number of large-scale (n > 100) clinical studies was small. Further meta-analysis evidence on MPC algorithm-based artificial pancreas systems using a large sample size and a long follow-up duration through individual trials is needed. Although it was confirmed that the MPC-based artificial pancreas system could statistically significantly improve glycemic control, heterogeneity existed due to age (especially adult or paediatric) [16, 55] or product type of intervention. To decrease heterogeneity, additional clinical trials on age or DIY systems are needed in the future.

## Conclusion

The aim of this study was to analyze whether MPC algorithm-based artificial pancreas systems were effective and safe for people with type 1 diabetes. The percentage of time maintained in the target blood glucose range was high in studies with overnight interventions. The percentage of time maintained in the hypoglycemic range was low in studies with overnight interventions when long follow-up period (more than 1 month) was considered.

## Supplementary Information

**Additional file 1. **“Risk of bias graph”. “Risk of bias summary”. “Subgroup analysis protocol”. “Mean difference in time maintained in the target blood glucose range according to the follow-up period (artificial pancreas (MPC-24h))”. “Mean difference in time maintained in the hypoglycemic range according to the follow-up period (artificial pancreas (MPC-24h))”. “Sensitivity analysis of only studies with a low risk of bias”. “Funnel plot of studies evaluating the percentage of time maintained in the target blood glucose range (3.9-10mmol)”. “Funnel plot of studies evaluating the percentage of time maintained in the hypoglycemic range (<3.9mmol)”. “Funnel plot of studies evaluating the daily insulin dose”. “PRISMA CheckList 2014”. “Detailed characteristic of the included studies”. “Mean difference in time maintained in the target blood glucose range and hypoglycemic range according to the timing of intervention and algorithm type”. “Mean difference in daily insulin dose(U) according to the intervention duration (overnight and 24h) and algorithm type (MPC, PID, and fuzzy) (artificial pancreas vs conventional insulin therapy)”. The supplementary figures show additional data analyzed by meta-analysis. The supplementary tables show the data reviewed systematically through the checklist, detailed study characteristics table, and additional meta-analysis data of primary and second outcomes.

## Data Availability

Not applicable.

## Abbreviations

MPC: Model Predictive Control
T1D: Type 1 diabetes
EMBASE: Excerpta medica data-BASE
PID: Proportional integral derivation
CSII: Continuous subcutaneous insulin infusion
SAP: Sensor augmented pump
CGM: Continuous glucose monitoring
FL: Fuzzy Logic
MeSH: Medical subject headings
PRISMA: Preferred reporting items for systematic review and meta-analyses
PROSPERO: International prospective register of systematic reviews
RCT: Randomized controlled trial
RoB: Risk of bias
MD: Mean difference
CI: Confidence intervals
IQR: Interquartile range
SD: Standard deviation
