# Supplementary material for: Effectiveness and safety of a model predictive control (MPC) algorithm for an artificial pancreas system in outpatients with type 1 diabetes (T1D): systematic review and meta-analysis
Source: Diabetol Metab Syndr. 2022 Dec 9;14:187. doi: 10.1186/s13098-022-00962-2 (PMC9733359; doi:10.1186/s13098-022-00962-2)
Supplement: Supplementary file 1 — Additional file 1. “Risk of bias graph”. “Risk of bias summary”. “Subgroup analysis protocol”. “Mean difference in time maintained in the target blood glucose range according to the follow-up period (artificial pancreas (MPC-24h))”. “Mean difference in time maintained in the hypoglycemic range according to the follow-up period (artificial pancreas (MPC-24h))”. “Sensitivity analysis of only studies with a low risk of bias”. “Funnel plot of studies evaluating the percentage of time maintained in the target blood glucose range (3.9-10mmol)”. “Funnel plot of studies evaluating the percentage of time maintained in the hypoglycemic range (<3.9mmol)”. “Funnel plot of studies evaluating the daily insulin dose”. “PRISMA CheckList 2014”. “Detailed characteristic of the included studies”. “Mean difference in time maintained in the target blood glucose range and hypoglycemic range according to the timing of intervention and algorithm type”. “Mean difference in daily insulin dose(U) according to the intervention duration (overnight and 24h) and algorithm type (MPC, PID, and fuzzy) (artificial pancreas vs conventional insulin therapy)”. The supplementary figures show additional data analyzed by meta-analysis. The supplementary tables show the data reviewed systematically through the checklist, detailed study characteristics table, and additional meta-analysis data of primary and second outcomes. [file 13098_2022_962_MOESM1_ESM.pdf]

Supplemental material

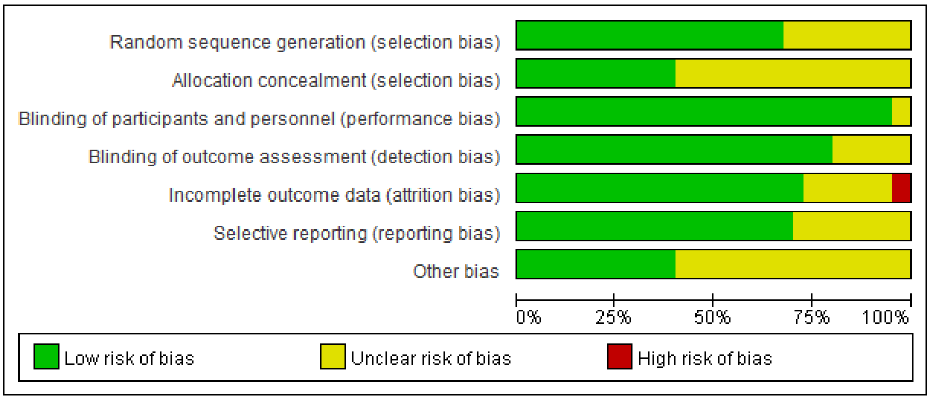

Supplementary Fig. 1 Risk of bias graph.

	Random sequence generation (selection bias)	Allocation concealment (selection bias)	Blinding of participants and personnel (performance bias)	Blinding of outcome assessment (detection bias)	Incomplete outcome data (attrition bias)	Selective reporting (reporting bias)	Other bias
Anderson et al	+	?	+	+	+	+	?
Bally et al	+	+	+	+	+	+	+
Benhamou et al	+	+	+	+	?	+	?
Blauw et al	?	?	+	?	?	?	?
Breton et al	+	?	+	+	?	?	?
Breton et al 2	?	?	+	+	+	?	+
Brown et al	+	+	+	+	?	?	?
Brown et al 2	+	?	+	?	+	+	?
Brown et al 3	+	?	+	+	+	+	?
Chernawsky et al	?	?	+	+	+	?	?
De bock et al	+	?	+	+	+	+	?
De boer et al	+	?	+	?	+	?	?
Del Favero et al	?	?	+	+	+	+	?
El-Khatib et al	+	+	+	+	+	+	+
Elleri et al	?	?	+	?	+	+	?
Forlenza et al	+	?	+	?	+	?	?
Forlenza et al 2	+	+	+	+	+	+	+
Haidar et al	+	+	+	+	+	+	+
Hovorka et al	+	+	+	+	+	+	+
Huyett et al	?	?	?	?	+	?	?
Kovatchev et al	+	+	+	+	+	+	+
Kovatchev et al 2	+	?	+	+	+	+	?
Kovatchev et al 3	+	?	+	+	+	+	+
Kropff et al	+	+	+	+	?	+	?
Leelarathna et al	+	+	+	+	+	+	+
Ly et al	?	?	+	+	?	?	?
Ly et al 2	?	?	+	?	+	+	+
Ly et al 3	?	?	+	+	−	?	?
Nimri et al	+	?	+	+	?	?	?
Nimri et al 2	+	?	+	+	−	?	?
Renard et al	?	?	+	+	?	+	?
Russell et al	?	?	+	+	+	+	?
Russell et al 2	?	?	+	+	?	+	?
Sherr et al	?	?	?	?	+	+	?
Spaic et al	+	+	+	+	+	+	+
Tauschmann et al	+	+	+	+	+	+	+
Tauschmann et al 2	+	+	+	+	+	+	+
Tauschmann et al 3	+	+	+	+	+	+	+
Thabit et al	+	+	+	+	+	+	+
Thabit et al 2	+	+	+	+	+	+	+

Supplementary Fig. 2 Risk of bias summary.

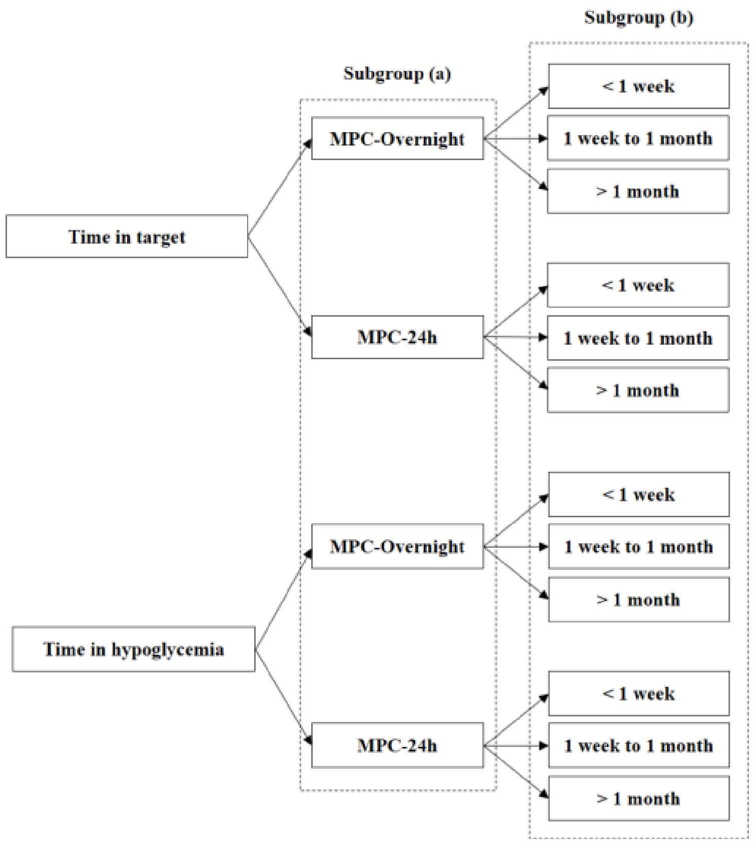

Supplementary Fig. 3 Subgroup analysis protocol. (a) intervention duration and (b) follow-up period.

	Artificial pancreas			Conventional therapy				Mean Difference
Study or Subgroup	Mean	SD	Total	Mean	SD	Total	Weight	IV, Random, 95% CI
**1.5.1 < 1 week**								
Breton et al 2	67.7	17.5	16	62.7	16.5	16	3.3%	5.00 [-6.79, 16.79]
Chernawsky et al	43.4	6.5	16	18.9	7	16	4.7%	24.50 [19.82, 29.18]
De boer et al	73.1	2.7	12	46.9	2.7	12	5.0%	26.20 [24.04, 28.36]
Del Favero et al	56.8	13.5	30	63.1	11	30	4.4%	-6.30 [-12.53, -0.07]
Huyett et al	71	10	10	57	16	10	3.4%	14.00 [2.31, 25.69]
Renard et al	63.8	8.9	23	48.5	13.6	23	4.4%	15.30 [8.66, 21.94]
Russell et al	80.6	7.4	19	57.6	14	19	4.3%	23.00 [15.88, 30.12]
Russell et al 2a	79.5	8.3	20	58.8	14.6	20	4.2%	20.70 [13.34, 28.06]
Russell et al 2b	75.9	7.9	32	64.5	14.1	32	4.5%	11.40 [5.80, 17.00]
**Subtotal (95% CI)**			**178**			**178**	**38.2%**	**15.18 [7.84, 22.51]**
Heterogeneity: Tau² = 111.84; Chi² = 121.02, df = 8 (P < 0.00001); I² = 93%								
Test for overall effect: Z = 4.06 (P < 0.0001)								
**1.5.2 1 week ~ 1 month**								
Anderson et al	77.9	11	21	59.3	15.7	21	4.1%	18.60 [10.40, 26.80]
Bally et al	76.2	6.4	29	65.6	8.1	28	4.8%	10.60 [6.80, 14.40]
De bock et al	74.32	8.41	12	75.82	8.49	12	4.3%	-1.50 [-8.26, 5.26]
El-khatib et al	78.4	6	39	61.9	14.4	39	4.7%	16.50 [11.60, 21.40]
Forlenza et al	71.6	9.8	19	65.2	10.4	19	4.4%	6.40 [-0.03, 12.83]
Haidar et al	69	15.6	36	61	9.63	36	4.5%	8.00 [2.01, 13.99]
Leelarathna et al	74.5	13.19	17	61.8	12.4	17	4.0%	12.70 [4.09, 21.31]
Tauschmann et al 2	66.6	7.9	12	47.7	14.4	12	3.8%	18.90 [9.61, 28.19]
Tauschmann et al 3	72	13.3	12	53	9.63	12	3.8%	19.00 [9.71, 28.29]
**Subtotal (95% CI)**			**197**			**196**	**38.4%**	**11.70 [7.49, 15.92]**
Heterogeneity: Tau² = 28.86; Chi² = 29.98, df = 8 (P = 0.0002); I² = 73%								
Test for overall effect: Z = 5.44 (P < 0.00001)								
**1.5.3 > 1 month**								
Benhamou et al	68.5	9.4	63	59.4	10.2	63	4.9%	9.10 [5.67, 12.53]
Breton et al	67	10	78	55	13	22	4.5%	12.00 [6.13, 17.87]
Brown et al	71	12	112	59	14	56	4.8%	12.00 [7.71, 16.29]
Tauschmann et al	65	8	46	54	9	40	4.8%	11.00 [7.38, 14.62]
Thabit et al	67.7	10.6	32	56.8	14.2	33	4.5%	10.90 [4.82, 16.98]
**Subtotal (95% CI)**			**331**			**214**	**23.4%**	**10.70 [8.78, 12.62]**
Heterogeneity: Tau² = 0.00; Chi² = 1.41, df = 4 (P = 0.84); I² = 0%								
Test for overall effect: Z = 10.94 (P < 0.00001)								
**Total (95% CI)**			**706**			**588**	**100.0%**	**12.99 [9.32, 16.67]**
Heterogeneity: Tau² = 69.22; Chi² = 238.75, df = 22 (P < 0.00001); I² = 91%								
Test for overall effect: Z = 6.93 (P < 0.00001)								
Test for subgroup differences: Chi² = 1.43, df = 2 (P = 0.49), I² = 0%								

Mean Difference
IV, Random, 95% CI

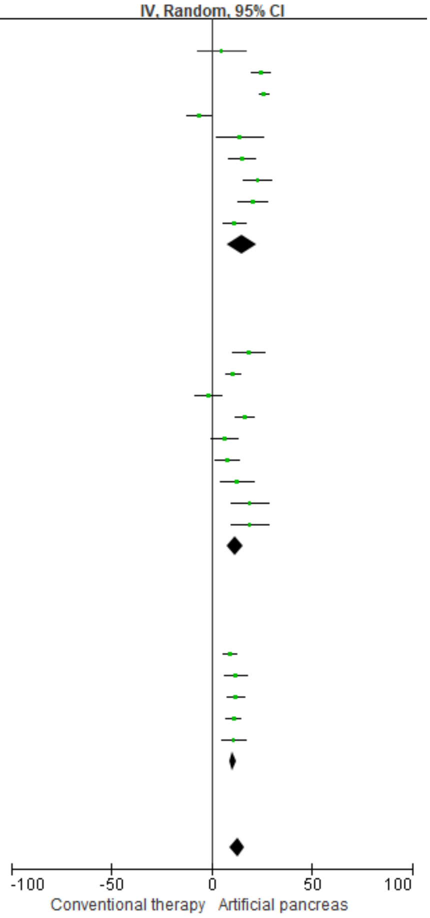

Supplementary Fig. 4 Mean difference in time maintained in the target blood glucose range according to the follow-up period (artificial pancreas (MPC-24h)).

Study or Subgroup	Artificial pancreas Mean	SD	Total	Conventional therapy Mean	SD	Total	Weight	Mean Difference IV, Random, 95% CI
**2.7.1 < 1 week**								
Breton et al 2	1.6	1.9	16	3.6	3.2	16	4.0%	-2.00 [-3.82, -0.18]
De boer et al	1.1	1.1	12	1.6	1.2	12	7.3%	-0.50 [-1.42, 0.42]
Del Favero et al	2	6.81	30	6.7	2.44	30	2.5%	-4.70 [-7.29, -2.11]
Huyett et al	2.5	0.8	10	4.2	3.1	10	3.6%	-1.70 [-3.68, 0.28]
Renard et al	2.1	3.78	23	3.1	1.63	23	4.4%	-1.00 [-2.68, 0.68]
Russell et al 2a	4.1	3.5	20	7.3	4.7	20	2.5%	-3.20 [-5.77, -0.63]
Russell et al 2b	3.1	2.7	32	4.9	5.1	32	3.6%	-1.80 [-3.80, 0.20]
**Subtotal (95% CI)**			**143**			**143**	**27.8%**	**-1.81 [-2.80, -0.82]**
Heterogeneity: Tau² = 0.88; Chi² = 12.66, df = 6 (P = 0.05); I² = 53%								
Test for overall effect: Z = 3.57 (P = 0.0004)								
**2.7.2 1 week ~ 1 month**								
Anderson et al	2.1	1.6	21	4.7	4.3	21	3.6%	-2.60 [-4.56, -0.64]
Bally et al	2.9	1.26	29	5.3	4.81	28	3.9%	-2.40 [-4.24, -0.56]
De bock et al	2.16	0.97	12	3.31	1.83	12	6.2%	-1.15 [-2.32, 0.02]
Forlenza et al	1.3	1.2	19	2.7	2.3	19	6.2%	-1.40 [-2.57, -0.23]
Haidar et al	1.6	1.19	36	3.5	3.4	36	6.2%	-1.90 [-3.08, -0.72]
Leelarathna et al	3.7	4.22	17	5	4.59	17	2.0%	-1.30 [-4.26, 1.66]
Tauschmann et al 2	4.3	2.81	12	2.4	4	12	2.2%	1.90 [-0.87, 4.67]
Tauschmann et al 3	2.9	2.2	12	1.7	3.1	12	3.2%	1.20 [-0.95, 3.35]
**Subtotal (95% CI)**			**158**			**157**	**33.7%**	**-1.20 [-2.06, -0.33]**
Heterogeneity: Tau² = 0.74; Chi² = 14.47, df = 7 (P = 0.04); I² = 52%								
Test for overall effect: Z = 2.71 (P = 0.007)								
**2.7.3 > 1 month**								
Benhamou et al	2	2.4	63	4.3	2.4	63	7.7%	-2.30 [-3.14, -1.46]
Breton et al	1.6	1.19	78	1.8	1.4	22	8.6%	-0.20 [-0.84, 0.44]
Brown et al	1.58	1.15	112	2.25	1.46	56	9.4%	-0.67 [-1.11, -0.23]
Tauschmann et al	2.6	1.26	46	3.9	2.67	40	7.4%	-1.30 [-2.20, -0.40]
Thabit et al	2.9	2.3	32	3	3.2	33	5.5%	-0.10 [-1.45, 1.25]
**Subtotal (95% CI)**			**331**			**214**	**38.5%**	**-0.93 [-1.65, -0.21]**
Heterogeneity: Tau² = 0.50; Chi² = 18.19, df = 4 (P = 0.001); I² = 78%								
Test for overall effect: Z = 2.53 (P = 0.01)								
**Total (95% CI)**			**632**			**514**	**100.0%**	**-1.25 [-1.72, -0.78]**
Heterogeneity: Tau² = 0.56; Chi² = 49.39, df = 19 (P = 0.0002); I² = 62%								
Test for overall effect: Z = 5.25 (P < 0.00001)								
Test for subgroup differences: Chi² = 1.98, df = 2 (P = 0.37), I² = 0%								

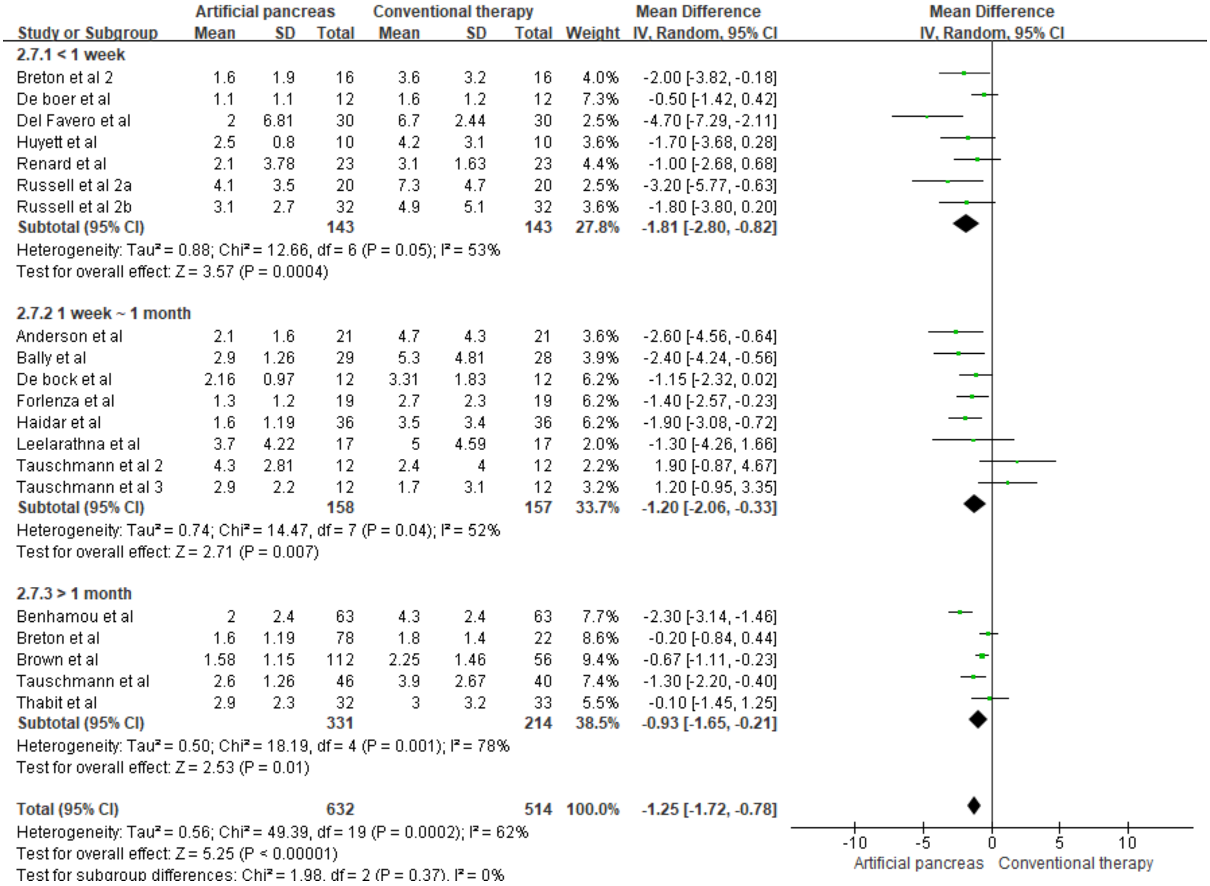

Supplementary Fig. 5 Mean difference in time maintained in the hypoglycemic range according to the follow-up period (artificial pancreas (MPC-24h)).

Study or Subgroup	Artificial pancreas Mean	SD	Total	Conventional therapy Mean	SD	Total	Weight	Mean Difference IV, Fixed, 95% CI
**1.7.1 Overnight**								
Forlenza et al 2	76	9	28	66	11	28	8.4%	10.00 [4.74, 15.26]
Hovoka et al	85	19.26	16	69	33.3	16	0.7%	16.00 [-2.85, 34.85]
Kovatchev et al	64	11	64	57	14	61	11.8%	7.00 [2.57, 11.43]
Spaic et al	78	10	30	71	10	30	9.1%	7.00 [1.94, 12.06]
Tauschmann et al 2	73.2	9	24	61.2	13.7	24	5.4%	12.00 [5.44, 18.56]
**Subtotal (95% CI)**			**162**			**159**	**35.3%**	**8.64 [6.08, 11.20]**
Heterogeneity: Chi² = 2.78, df = 4 (P = 0.60); I² = 0%								
Test for overall effect: Z = 6.61 (P < 0.00001)								
**1.7.2 24h**								
Bally et al	76.2	6.4	29	65.6	8.1	28	16.1%	10.60 [6.80, 14.40]
El-khatib et al	78.4	6	39	61.9	14.4	39	9.7%	16.50 [11.60, 21.40]
Haidar et al	69	15.6	36	61	9.63	36	6.5%	8.00 [2.01, 13.99]
Leelarathna et al	74.5	13.19	17	61.8	12.4	17	3.1%	12.70 [4.09, 21.31]
Tauschmann et al	65	8	46	54	9	40	17.7%	11.00 [7.38, 14.62]
Tauschmann et al 2	66.6	7.9	12	47.7	14.4	12	2.7%	18.90 [9.61, 28.19]
Tauschmann et al 3	72	13.3	12	53	9.63	12	2.7%	19.00 [9.71, 28.29]
Thabit et al	67.7	10.6	32	56.8	14.2	33	6.3%	10.90 [4.82, 16.98]
**Subtotal (95% CI)**			**223**			**217**	**64.7%**	**12.16 [10.26, 14.05]**
Heterogeneity: Chi² = 10.20, df = 7 (P = 0.18); I² = 31%								
Test for overall effect: Z = 12.58 (P < 0.00001)								
**Total (95% CI)**			**385**			**376**	**100.0%**	**10.92 [9.39, 12.44]**
Heterogeneity: Chi² = 17.65, df = 12 (P = 0.13); I² = 32%								
Test for overall effect: Z = 14.05 (P < 0.00001)								
Test for subgroup differences: Chi² = 4.67, df = 1 (P = 0.03), I² = 78.6%								

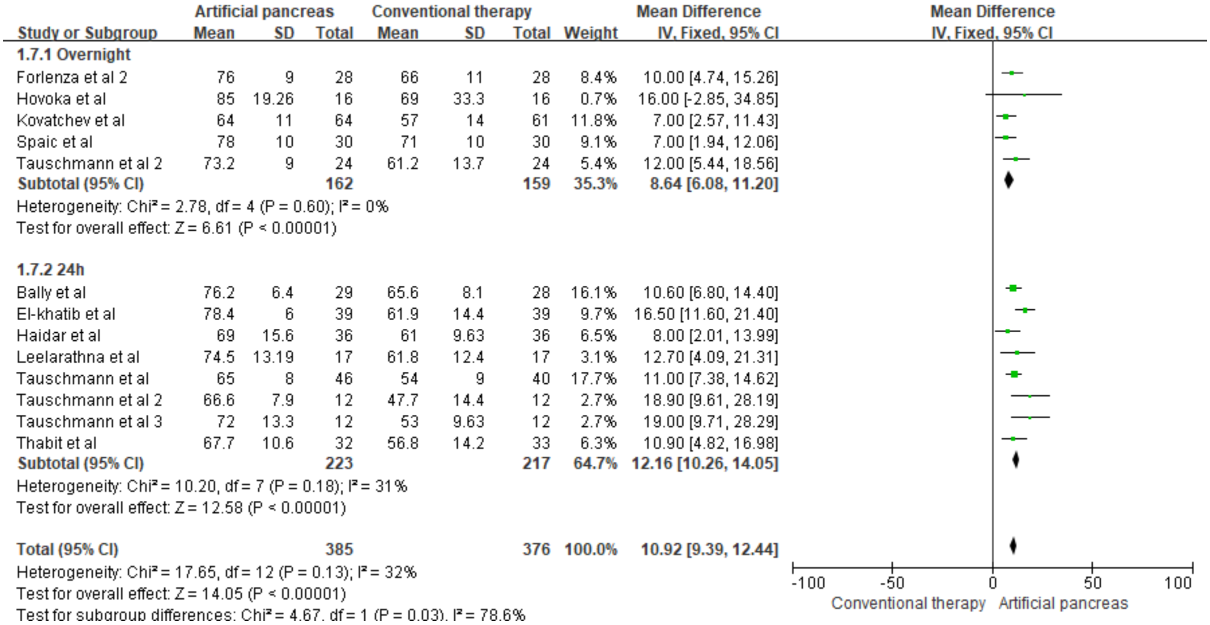

Supplementary Fig. 6 Sensitivity analysis of only studies with a low risk of bias.

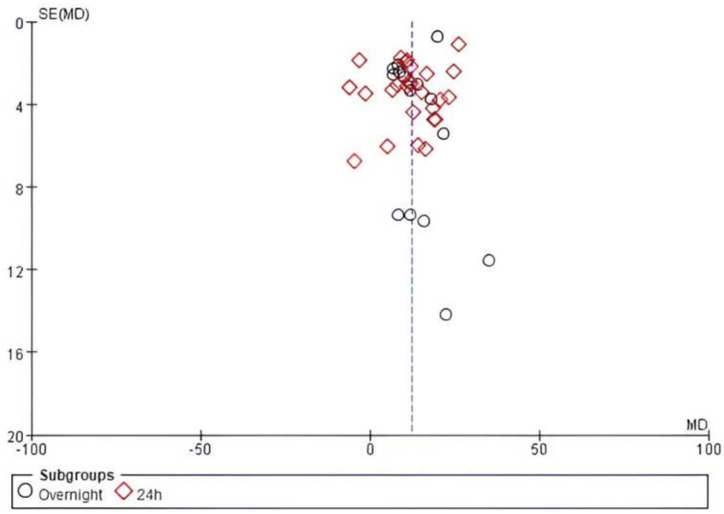

Supplementary Fig. 7 Funnel plot of studies evaluating the percentage of time maintained in the target blood glucose range (3.9-10mmol).

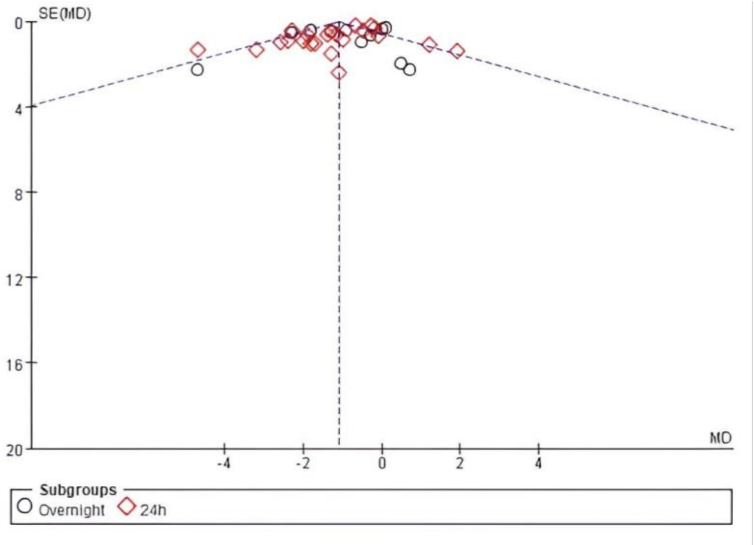

Supplementary Fig. 8 Funnel plot of studies evaluating the percentage of time maintained in the hypoglycemic range (<3.9mmol).

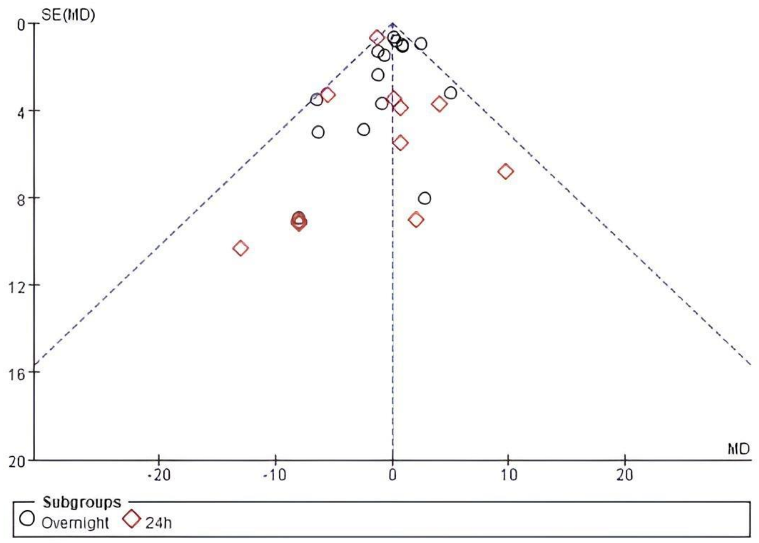

Supplementary Fig. 9 Funnel plot of studies evaluating the daily insulin dose.

Supplementary Table 1 PRISMA CheckList 2014.

Section and Topic	Item #	Checklist item	Location where item is reported
**TITLE**			
Title	1	Identify the report as a systematic review.	1
**ABSTRACT**			
Abstract	2	See the PRISMA 2020 for Abstracts checklist.	1
**INTRODUCTION**			
Rationale	3	Describe the rationale for the review in the context of existing knowledge.	1-2
Objectives	4	Provide an explicit statement of the objective(s) or question(s) the review addresses.	1-2
**METHODS**			
Eligibility criteria	5	Specify the inclusion and exclusion criteria for the review and how studies were grouped for the syntheses.	2
Information sources	6	Specify all databases, registers, websites, organisations, reference lists and other sources searched or consulted to identify studies. Specify the date when each source was last searched or consulted.	2
Search strategy	7	Present the full search strategies for all databases, registers and websites, including any filters and limits used.	2
Selection process	8	Specify the methods used to decide whether a study met the inclusion criteria of the review, including how many reviewers screened each record and each report retrieved, whether they worked independently, and if applicable, details of automation tools used in the process.	2
Data collection process	9	Specify the methods used to collect data from reports, including how many reviewers collected data from each report, whether they worked independently, any processes for obtaining or confirming data from study investigators, and if applicable, details of automation tools used in the process.	2
Data items	10a	List and define all outcomes for which data were sought. Specify whether all results that were compatible with each outcome domain in each study were sought (e.g. for all measures, time points, analyses), and if not, the methods used to decide which results to collect.	3
	10b	List and define all other variables for which data were sought (e.g. participant and intervention characteristics, funding sources). Describe any assumptions made about any missing or unclear information.	3
Study risk of bias assessment	11	Specify the methods used to assess risk of bias in the included studies, including details of the tool(s) used, how many reviewers assessed each study and whether they worked independently, and if applicable, details of automation tools used in the process.	3
Effect measures	12	Specify for each outcome the effect measure(s) (e.g. risk ratio, mean difference) used in the synthesis or presentation of results.	3
Synthesis methods	13a	Describe the processes used to decide which studies were eligible for each synthesis (e.g. tabulating the study intervention characteristics and comparing against the planned groups for each synthesis (item #5)).	3

Section and Topic	Item #	Checklist item	Location where item is reported
	13b	Describe any methods required to prepare the data for presentation or synthesis, such as handling of missing summary statistics, or data conversions.	3
	13c	Describe any methods used to tabulate or visually display results of individual studies and syntheses.	3
	13d	Describe any methods used to synthesize results and provide a rationale for the choice(s). If meta-analysis was performed, describe the model(s), method(s) to identify the presence and extent of statistical heterogeneity, and software package(s) used.	3
	13e	Describe any methods used to explore possible causes of heterogeneity among study results (e.g. subgroup analysis, meta-regression).	3
	13f	Describe any sensitivity analyses conducted to assess robustness of the synthesized results.	5
Reporting bias assessment	14	Describe any methods used to assess risk of bias due to missing results in a synthesis (arising from reporting biases).	3
Certainty assessment	15	Describe any methods used to assess certainty (or confidence) in the body of evidence for an outcome.	3
**RESULTS**			
Study selection	16a	Describe the results of the search and selection process, from the number of records identified in the search to the number of studies included in the review, ideally using a flow diagram.	Fig. 1
	16b	Cite studies that might appear to meet the inclusion criteria, but which were excluded, and explain why they were excluded.	3
Study characteristics	17	Cite each included study and present its characteristics.	Table 1
Risk of bias in studies	18	Present assessments of risk of bias for each included study.	Supple Fig. 1-2
Results of individual studies	19	For all outcomes, present, for each study: (a) summary statistics for each group (where appropriate) and (b) an effect estimate and its precision (e.g. confidence/credible interval), ideally using structured tables or plots.	Fig. 2-6
Results of syntheses	20a	For each synthesis, briefly summarise the characteristics and risk of bias among contributing studies.	3
	20b	Present results of all statistical syntheses conducted. If meta-analysis was done, present for each the summary estimate and its precision (e.g. confidence/credible interval) and measures of statistical heterogeneity. If comparing groups, describe the direction of the effect.	4
	20c	Present results of all investigations of possible causes of heterogeneity among study results.	4-5
	20d	Present results of all sensitivity analyses conducted to assess the robustness of the synthesized results.	Supple Fig. 4

Section and Topic	Item #	Checklist item	Location where item is reported
Reporting biases	21	Present assessments of risk of bias due to missing results (arising from reporting biases) for each synthesis assessed.	Supple Fig. 1-2
Certainty of evidence	22	Present assessments of certainty (or confidence) in the body of evidence for each outcome assessed.	4
**DISCUSSION**			
Discussion	23a	Provide a general interpretation of the results in the context of other evidence.	5
	23b	Discuss any limitations of the evidence included in the review.	6
	23c	Discuss any limitations of the review processes used.	6
	23d	Discuss implications of the results for practice, policy, and future research.	6
**OTHER INFORMATION**			
Registration and protocol	24a	Provide registration information for the review, including register name and registration number, or state that the review was not registered.	NA
	24b	Indicate where the review protocol can be accessed, or state that a protocol was not prepared.	NA
	24c	Describe and explain any amendments to information provided at registration or in the protocol.	NA
Support	25	Describe sources of financial or non-financial support for the review, and the role of the funders or sponsors in the review.	6
Competing interests	26	Declare any competing interests of review authors.	6
Availability of data, code and other materials	27	Report which of the following are publicly available and where they can be found: template data collection forms; data extracted from included studies; data used for all analyses; analytic code; any other materials used in the review.	6-10

Supplementary Table 2 Detailed characteristics of the included studies.

No	Study	Year	Participants (n)	Participants Characteristics	Artificial pancreas device components	Artificial pancreas algorithm	Comparator	24h or overnight	Follow-up	Hormone	Setting
1	Anderson et al [24].	2019	42	Mean age 38 years(3.3), TDD 49 units(4.8), HbA1c 7.2% (0.2; 55mmol/mol)	DiAs USS with Dexcom	MPC	SAP	24h	4 weeks	Single	Home
2	Bally et al [25].	2017	29	Mean age 41 years(13), TDD 0.5 units/kg(0.1), HbA1c 6.9% (0.5; 51.7mmol/mol)	Florence with FreeStyle Navigator	MPC	SAP	24h	4 weeks	Single	Home
3	Benhamou et al [26].	2019	63	Mean age 48.2 years (13.4), HbA1c 7.6% (0.9; 59.4mmol/mol)	Hybrid closed-loop system	MPC	SAP	24h	12 weeks	Single	Home
4	Blauw et al [27].	2016	10	Mean age 41 years (26·5–52·3), HbA1c 7·7% (7·4–8; 60·7 mmol/mol)	Inreda Diabetic	PID	Pump	24h	4 days	Dual	Home
5	Breton et al [28].	2020	101	Mean age 11.3 years(6-13), TDD 0.89units/kg(0.24), HbA1c 7.7% (1.1)	t:slim X2 insulin pump, Dexcom with Control-IQ Technology	MPC	SAP	24h	16 weeks	Single	Outpatient
6	Breton et al 2 [29].	2017	32	Mean age 13.2 years (10-16), TDD 0.9 units/kg (0.18), HbA1c 8.5% (1.5)	t:AP pump or Roche Accu-Chek Spirit Combo pump, Dexcom with DiAs	MPC	SAP	24h	120h	Single	Camp
7	Brown et al [30].	2019	168	Mean age 33 years (16), HbA1c 7.6% (0.8)	t:slim X2 insulin pump with Control-IQ Technology, Tandem Diabetes Care, Dexcom	MPC	SAP	24h	6 months	Single	Home
8	Brown et al 2 [31].	2017	40	Mean age 45.5 years (21-65), TDD 0.4units/kg(0.11), HbA1c 7.4% (0.8)	DiAs USS with Dexcom	MPC	SAP	Overnight	5 days	Single	Research house or hotel
9	Brown et al 3 [32].	2015	10	Mean age 46·8 years (8·5), TDD 0·4 units/kg (0·1), HbA1c 7·01% (1·05; 53.1mmol/mol)	Accu-Chek Spirit Combo pump or personal pump, Dexcom with DiAs system	PID	SAP	Overnight	5 days	Single	Research house or hotel
10	Chernavvsky et al [33].	2016	16	Mean age 15.2 years (13-17), HbA1c 8.2% (6.9-9.8)	DiAs USS with Dexcom	MPC	Pump	24h	1 day	Single	Research house
11	De bock et al [34].	2018	12	Mean age 15 years (13-17), HbA1c 8.55%	Medtronic MiniMed Hybrid Closed Loop System	MPC	Pump	24h	7 days	Single	Camp

No	Study	Year	Participants (n)	Participants Characteristics	Artificial pancreas device components	Artificial pancreas algorithm	Comparator	24h or overnight	Follow-up	Hormone	Setting
12	De boer et al [35].	2017	12	Mean age 7 years (0.37), TDD 0.75 units/kg(0.18), HbA1c 7.7% (0.52)	DiAs USS with Dexcom	MPC	SAP	24h	3 days	Single	Hotel or home
13	Del Favero et al [36].	2016	30	Mean age 7·6 years (1·2), TDD 0·78 units/kg (0·16), HbA1c 7·3% (0·9; 56·3 mmol/mol)	Accu-Chek Spirit Combo pump or personal pump, Dexcom with DiAs system	MPC	SAP	24h	72h	Single	Camp
14	El-Khatib et al [37].	2017	39	Mean age 33·3 years (11·1), TDD 0·6 units/kg (0·14), HbA1c 7·7% (1·2; 60·7 mmol/mol)	Two(one for insulin, one for glucagon) t:Slim infusion pumps, Dexcom	MPC	Pump	24h	11 days	Dual	Home
15	Elleri et al [38].	2013	12	Mean age 15 years (12-18), TDD 0.9 units/kg(0.3), HbA1c 7.9% (0.7)	SEVEN PLUS; Dexcom	MPC	Pump	Overnight	36h	Single	Outpatient
16	Forlenza et al [39].	2017	19	Mean age 23 years (10), TDD 0.67 units/kg(0.19), HbA1c 8% (1.7; 63.8mmol/mol)	DiAs	MPC	SAP	24h	2 weeks	Single	Home
17	Forlenza et al 2 [40].	2017	28	Mean age 12 years (6-14), TDD 0.83 units/kg(0.14), HbA1c 7.6% (1.1; 60mmol/mol)	Medtronic PHHM	MPC	SAP	Overnight	21 nights	Single	Home
18	Haidar et al [20].	2021	36	Mean age 39 years(16), TDD 0.65 units/kg (0.22), HbA1c 7.5% (0.8)	Dexcom CGM system, t:slim TAP3 insulin pump	MPC	SAP	24h	12 days	Single	Outpatient
19	Hovorka et al [41].	2014	16	Mean age 15·6 years (2·1),TDD 0·8 (0·2), HbA1c 8·0% (0·9)	Florence with FreeStyle Navigator	MPC	SAP	Overnight	21 days	Single	Home
20	Huyett et al [42].	2017	10	Mean age 15.3 years (11.9-17.7), TDD 0.82 units/kg(0.60-1.14), HbA1c 8.1% (1.3; 65mmol/mol)	DiAs with Dexcom	MPC	SAP	24h	72h	Single	Outpatient
21	Kovatchev et al [43].	2020	125	Mean age 33 years (14-70), HbA1c 7.4% (0.9; 57mmol/mol)	Accu-Chek Spirit Combo insulin pump, Dexcom CGM system, and inControlAP	MPC	SAP	Overnight	3 months	Single	Outpatient

No	Study	Year	Participants (n)	Participants Characteristics	Artificial pancreas device components	Artificial pancreas algorithm	Comparator	24h or overnight	Follow-up	Hormone	Setting
22	Kovatchev et al 2 [44].	2020	78	Mean age 42.3 years (11.9), HbA1c 7.42% (1.03)	Accu-Chek Spirit Combo insulin pump, Dexcom CGM system, and inControlAP	MPC	SAP	Overnight	10 months	Single	Outpatient
23	Kovatchev et al 3 [45].	2014	18	Mean age 46 years (10), HbA1c 7·4% (0·7; 57·4 mmol/mol)	Tandem t:slim pump, with DiAs system	PID	SAP	24h	40h	Single	Hotel or guesthouse
24	Kropff et al [46].	2015	32	Mean age 47 years (11·2), TDD 0·6 units/kg (0·1), HbA1c 8·2% (0·6;66·1mmol/mol)	Accu-Chek Spirit Combo insulin pump, Dexcom CGM system	MPC	SAP	Overnight	12 weeks	Single	Home
25	Leelarathna et al [47].	2014	17	Mean age 34 years (9), TDD 0·53 units/kg (0·12), HbA1c 7·6% (0·8; 59·6 mmol/mol)	Florence with FreeStyle Navigator	MPC	SAP	24h	8 days	Single	Home
26	Ly et al [48].	2016	21	Mean age 14·7 years (3·9), TDD 0·8 units/kg (0·2), HbA1c 7·9% (1·4; 62·8 mmol/mol)	Medtronic MiniMed Hybrid Closed Loop System	PID	SAP	Overnight	5-6 days	Single	Camp
27	Ly et al 2 [49].	2015	21	Mean age 18·6 years (3·7), TDD 0·8 units/kg (0·2), HbA1c 8·6% (1·5; 70·5 mmol/mol)	Medtronic MiniMed Hybrid Closed Loop System	PID	SAP	24h	6 days	Single	Camp
28	Ly et al 3 [50].	2014	20	Mean age 15·3 years (2·9), HbA1c 8·1% (1·1; 65 mmol/mol)	Medtronic MiniMed Hybrid Closed Loop System	PID	SAP	Overnight	5-6 days	Single	Camp
29	Nimri et al [51].	2014	24	Mean age 21·2 years (8·9), TDD 0·8 units/kg (0·3), HbA1c 8·5% (0·8; 69·4 mmol/mol)	MD-Logic system with Medtronic Paradigm Veo pump	Fuzzy	SAP	Overnight	6 weeks	Single	Home
30	Nimri et al 2 [52].	2014	15	Mean age 19 years (10·4), TDD 0·9 units/kg (0·3), HbA1c 7·5% (0·5; 58·5mmol/mol)	MD-Logic system with Medtronic Paradigm Veo pump	Fuzzy	SAP	Overnight	4 days	Single	Home
31	Renard et al [53].	2018	23	Mean age 9.4 years (7-12), TDD 0.8 units/kg (0.2), HbA1c 7.5% (0.5; 58mmol/mol)	DiAs with Dexcom	MPC	SAP	24h	2 days	Single	Outpatient

No	Study	Year	Participants (n)	Participants Characteristics	Artificial pancreas device components	Artificial pancreas algorithm	Comparator	24h or overnight	Follow-up	Hormone	Setting
32	Russell et al [54].	2016	19	Mean age 9·8 years (1·6), TDD 0·74 units/kg (0·15), HbA1c 7·8% (0·8; 61·7 mmol/mol)	Two(one for insulin, one for glucagon) t:Slim infusion pumps, Dexcom	MPC	Pump	24h	5 days	Dual	Camp
33	Russell et al 2a [55].	2014	20	Mean age 40 years (16), TDD 0·5 units/kg (0·11), HbA1c 7·1% (0·8; 4·1 mmol/mol)	Two(one for insulin, one for glucagon) t:Slim infusion pumps, Dexcom	MPC	Pump	24h	5 days	Dual	Home
34	Russell et al 2b [55].	2014	32	Mean age 16 years (3), TDD 0·8 units/kg (0·18), HbA1c 8.2%(1; 66.1mmol/mol)	Two(one for insulin, one for glucagon) t:Slim infusion pumps, Dexcom	MPC	Pump	24h	5 days	Dual	Camp
35	Sherr et al [56].	2020	11	Mean age 28.8 years (7.9), HbA1c 7.4% (1.2)	Omnipod hybrid closed loop system	MPC	Pump	Overnight	7 days	Single	Hotel or home
36	Spaic et al [57].	2017	30	Mean age 31 years (15-43), TDD 0.58 units/kg (0.16), HbA1c 7.1% (0.59; 54mmol/mol)	Medtronic PHHM	MPC	SAP	Overnight	21 nights	Single	Home
37	Tauschmann et al [58].	2018	86	Mean age 22 years (13-36), TDD 0.76 units/kg (0.25), HbA1c 8.3% (0.6)	Medtronic Hybrid Closed Loop System	MPC	SAP	24h	12 weeks	Single	Outpatient
38	Tauschmann et al 2 [59].	2016	12	Mean age 14·6 years (3·1), TDD 0·82 units/kg (0·18), HbA1c 8·5% (0·7; 69·4 mmol/mol)	Florence with FreeStyle Navigator	MPC	SAP	24h	3 weeks	Single	Home
39	Tauschmann et al 3 [60].	2016	12	Mean age 15·4 years (2·6), TDD 0·84 units/kg (0·22), HbA1c 8·3% (0·9; 67·2 mmol/mol)	Florence with FreeStyle Navigator	MPC	SAP	24h	7 days	Single	Home
40	Thabit et al [61].	2015	33	Mean age 40 years (9·4), TDD 0·62 units/kg (0·15), HbA1c 8·5% (0·7; 69·4 mmol/ mol)	Florence with FreeStyle Navigator	MPC	SAP	24h	12 weeks	Single	Home
41	Thabit et al 2 [62].	2014	24	Mean age 43 years (12), TDD 0·5 units/kg (0·1), HbA1c 8·1% (0·8; 65 mmol/mol)	Florence with FreeStyle Navigator	MPC	SAP	Overnight	4 weeks	Single	Home

MPC = Model Predictive Control, PID = Proportional Integral Derivative, SAP = Sensor-Augmented Pump

TDD = Total Daily Dose, BMI = Body Mass Index, CGM = Continuous Glucose Monitoring, DiAs = Diabetes Assistant

Supplementary Table 3 Mean difference in time maintained in the target blood glucose range and hypoglycemic range according to the timing of the intervention and algorithm type.

	Number of comparisons	Mean difference (95% CI)	p value	I^2
**Percentage of time maintained in the target blood glucose range**				
All comparisons	41	12.56 (9.80, 15.31)	<0.00001	90%
Intervention duration				
Overnight	15	13.04 (9.05, 17.04)	<0.00001	84%
24h	26	11.88 (7.99, 15.78)	<0.00001	93%
Algorithm				
MPC vs Conventional therapy	33	12.57 (9.63, 15.50)	<0.00001	89%
PID vs Conventional therapy	6	9.59 (-3.67, 22.85)	<0.00001	96%
Fuzzy vs Conventional therapy	2	16.52 (9.72, 23.32)	0.23	29%
**Percentage of time maintained in the hypoglycemic blood glucose range**				
All comparisons	35	-1.62 (-2.43, -0.81)	<0.00001	94%
Intervention duration				
Overnight	13	-2.39 (-4.61, -0.18)	<0.00001	98%
24h	22	-1.16 (-1.59, -0.73)	<0.00001	63%
Algorithm				
MPC vs Conventional therapy	30	-1.12 (-1.50, -0.75)	<0.00001	64%
PID vs Conventional therapy	3	-5.24 (-16.06, 5.58)	<0.00001	100%
Fuzzy vs Conventional therapy	2	-20.80 (-64.12, 22.52)	0.0007	91%

CI=Confidence Intervals, MPC=Model Predictive Control, PID=Proportional Integral Derivative

Supplementary Table 4 Mean difference in daily insulin dose(U) according to the intervention duration (overnight and 24h) and algorithm type (MPC, PID, and fuzzy) (artificial pancreas vs conventional insulin therapy).

	Number of comparisons	Mean difference (95% CI)	P value	I^2
**All comparisons**	26	0.13 (0.02, 0.24)	0.02	26%
	**Overnight**		**24h**	
	**Number of comparisons**	**Mean difference (95% CI; p value)**	**Number of comparisons**	**Mean difference (95% CI; p value)**
**MPC** vs Conventional therapy	7	0.02 (-1.77, 1.82; p=0.98)	9	-1.24 (-2.43, -0.06; p=0.04)
**PID** vs Conventional therapy	4	0.10 (-1.11, 1.31; p=0.87)	2	1.85 (-15.38, 19.08; p=0.83)
**Fuzzy** vs Conventional therapy	2	-1.19 (-3.46, 1.08; p=0.30)	-	-

CI=Confidence Intervals, MPC=Model Predictive Control, PID=Proportional Integral Derivative
